# Fabrication and Characterization of a Flexible Fluxgate Sensor with Pad-Printed Solenoid Coils†

**DOI:** 10.3390/s20082275

**Published:** 2020-04-16

**Authors:** Spyridon Schoinas, Adyl-Michaël El Guamra, Fabien Moreillon, Philippe Passeraub

**Affiliations:** Institute of Industrial Technologies and Sciences, University of Applied Sciences and Arts Western Switzerland (HES-SO Geneva/HEPIA), CH-1202 Geneva, Switzerland; a.elguamra@meniconrd.com (A.-M.E.G.); fabien.moreillon@hesge.ch (F.M.); philippe.passeraub@hesge.ch (P.P.)

**Keywords:** fluxgate sensor, Solenoid coils, magnetic sensor, magnetometer, pad-printing, flexible electronics, additive fabrication process

## Abstract

This paper presents the fabrication and characterization of a flexible, flat, miniaturized fluxgate sensor with a thin amorphous rectangular magnetic core fabricated by the pad/printing technique. Both the design and the various printing steps of the sensor are presented. The fluxgate sensor comprises of solenoid coils, and to the best of our knowledge, is the first to be printed with a conventional micro-printing technique. The magnetic core is a non-printed component, placed between the printed layers. The sensor’s linear measuring range is ±40 µT with 2% full-scale linearity error, at 100 kHz excitation frequency. The highest measured sensitivity reaches 14,620 V/T at 200 kHz, while the noise of the sensor was found to be 10 nT/$\sqrt{\mathrm{Hz}}$ at 1 Hz.

## 1. Introduction

Fluxgate sensors are magnetic flux density measurement instruments. They are high-accuracy direction sensitive magnetometers that include the Earth’s magnetic field in their measuring range. For this reason, they are commonly used for magnetic compass applications. Among others, many current measurement devices are based on fluxgate sensors, since they can measure DC magnetic fields as well as low-frequency AC magnetic fields [1,2]. Since a high resolution and precision are less important features in consumer electronic devices, fluxgate sensors have been replaced by anisotropic magnetoresistance (AMR) sensors due to their complicated fabrication techniques [1]. Nevertheless, printed circuit board (PCB) technology [3,4,5,6,7,8], glass microfabrication technology [9,10] and other silicon-based microfabrication technologies [11,12,13] have introduced miniaturized fluxgate sensors, fabricated with simplified techniques.

Recent developments in various micro-printing techniques allow fluxgate sensors and other microfabricated devices to obtain additional characteristics such as flexibility, ease of integration and eco-friendliness. Micro-printing techniques include inkjet printing, aerosol jet printing, screen printing, gravure printing, flexographic printing and offset printing. A detailed review of these techniques is presented in [14,15,16,17]. Despite their low resolution compared to clean-room techniques, micro-printing techniques can provide relatively fast and simple production methods along with the ease of industrialization. These attractive characteristics can be fully exploited in applications in the domains of the Internet of Things (IoT), wearables and medical devices. Micro-printed devices have already been introduced to market in the form of Radio-frequency identification (RFID) tags, displays, electrodes, and sensors [17,18,19,20,21]. These devices are known as printed electro-mechanical systems (PEMS) [21].

The fabrication of the sensor presented in this paper is based on the pad-printing technique. The pad-printing technique is an off-set printing technique, which is widely used in the watchmaking industry for the creation of watch-faces. The principles of the fabrication process are explained in [14,21,22,23]. The standard printing cycle is composed of the following steps: the ink cup is filled with ink to produce the motif on the engraved plate. Then, the pad (silicon rubber stamp) transfers the motif of ink onto the substrate.

For the creation of simple sensors, electrodes and conductive tracks, a specific process has been developed which meets the standards of technology readiness level (TRL) 4; thus, the creation of these systems is validated in the laboratory. The biggest advantages of this process compared to other printing techniques are the high printing rate (>1500 prints/h), the ability to use volatile solvents that allow fast drying of the motif onto the substrate, the adaptability of this technique to output range from a small number of fabrication up to mass production, the precise layer-by-layer printing, and the ability to print on non-flat surfaces [14]. All these features made the inclusion of the soft magnetic core in the process possible—an otherwise non-printed component.

The flexibility of magnetoimpedance (MI) sensors and giant magnetoresistance (GMR) sensors has been studied in [24] and [25], respectively. Previously reported flexible solenoid coils obtained via the screen-printing technique are presented in [26]. However, the fluxgate sensor presented in this paper is the first to be fabricated using a simple micro-printing technique. To the best of our knowledge, this study reports the first solenoid coils to be obtained through the use of a pad printing technique. This presents the potential development of micro-transformers via micro-printing techniques. In this paper, we present the fabrication process of the developed fluxgate sensor, study its performance and discuss its properties.

## 2. Materials and Methods

### 2.1. Materials

“SpectraZ 14” silver-based inks, specially developed by the Laboratory of Microengineering and Bioinstrumentation of the Institute of Industrial Technologies and Sciences from the University of Applied Sciences and Arts Western Switzerland, were used for the conductive tracks. “Teca-Print TPC 528” (Teca-Print AG, Thayngen, Switzerland) commercial resin was used for the insulation layers of the sensor. “Vitrovac 6025Z” (VACUUMSCHMELZE GmbH & Co. KG, Hanau, Germany) in the form of 25 μm-thick ribbon was selected for the soft magnetic core of the sensor. “Kapton^®^” (DuPont, Wilmington, CT, USA) polyamide film with 127 μm thickness was used as a substrate. “UHU^®^ plus quick safe” (UHU GmbH & Co., Bühl, Germany) two-component epoxy-based glue was used for the fixation of the magnetic core on the insulating layer.

### 2.2. Sensor Design and Fabrication

**Figure 1:**
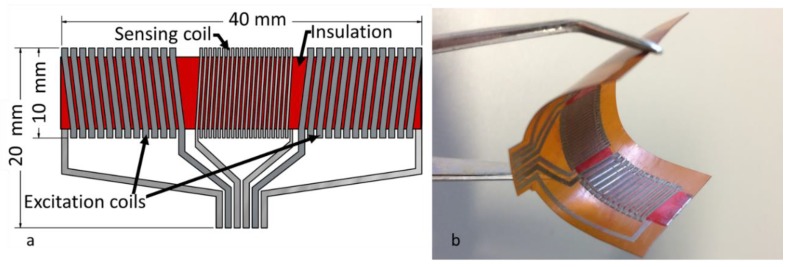
Fluxgate sensor: (**a**) Schematic representation of the conception of the sensor with dimensions; (**b**) Photographic view of the fabricated flexible sensor.

A simplified “bar sensor” design was adopted to facilitate the fabrication process. The design of the sensor is shown in Figure 1. The sensor, first presented in [27], comprises three solenoid coils, the magnetic core and the insulation that surrounds the core. From these three coils, the one in the middle is used as a sensing coil with 21 turns, and the others as excitation coils with 13 turns each. Furthermore, the role of the insulation is to prevent short-circuits between the magnetic core and the coils. The dimensions of the entire sensor are 40 mm × 20 mm × 0.3 mm, while the width of the solenoid coils is 10 mm. Moreover, the dimensions of the magnetic core are 40 mm × 7 mm × 0.025 mm. The conductive track width of the sensing coils is 250 μm and the spacing between those conductive tracks is the same. The track width of the excitation coils is 650 μm, while the spacing between the conductive tracks is 350 μm.

The most important component of the sensor is the soft magnetic core. Vitrovac 6025Z was selected because of its very high magnetic permeability and low magnetic noise properties due to its amorphous state. The ideal magnetic core should mirror the “Solid-liquid” model discussed in [28], where the magnetic domains can move freely without any friction. Any attempt based on this model should minimize the noise of the device. Moreover, the noise of the sensor does not only depend on the soft magnetic material but also on the shape of the core [1]. The bar sensor, with its single-core design, has a similar behavior to that of a transformer. Therefore, high noise levels are expected for this sensor design.

The fabrication of the sensor does not require a cleanroom. However, to achieve a high standard of repetitive printing of fine patterns, a stable temperature and humid environment is necessary for the pad-printing process due to the required control of ink properties. The average thickness of the printed layer depends on the combination of the etching depth of the engraved plate, the viscosity of the inks and the adhesion properties of the pad and substrate. The average thickness of each print is 3 µm, which is relatively low compared to the etching depth of the engraved plate that reaches 20 µm.

**Figure 2:**
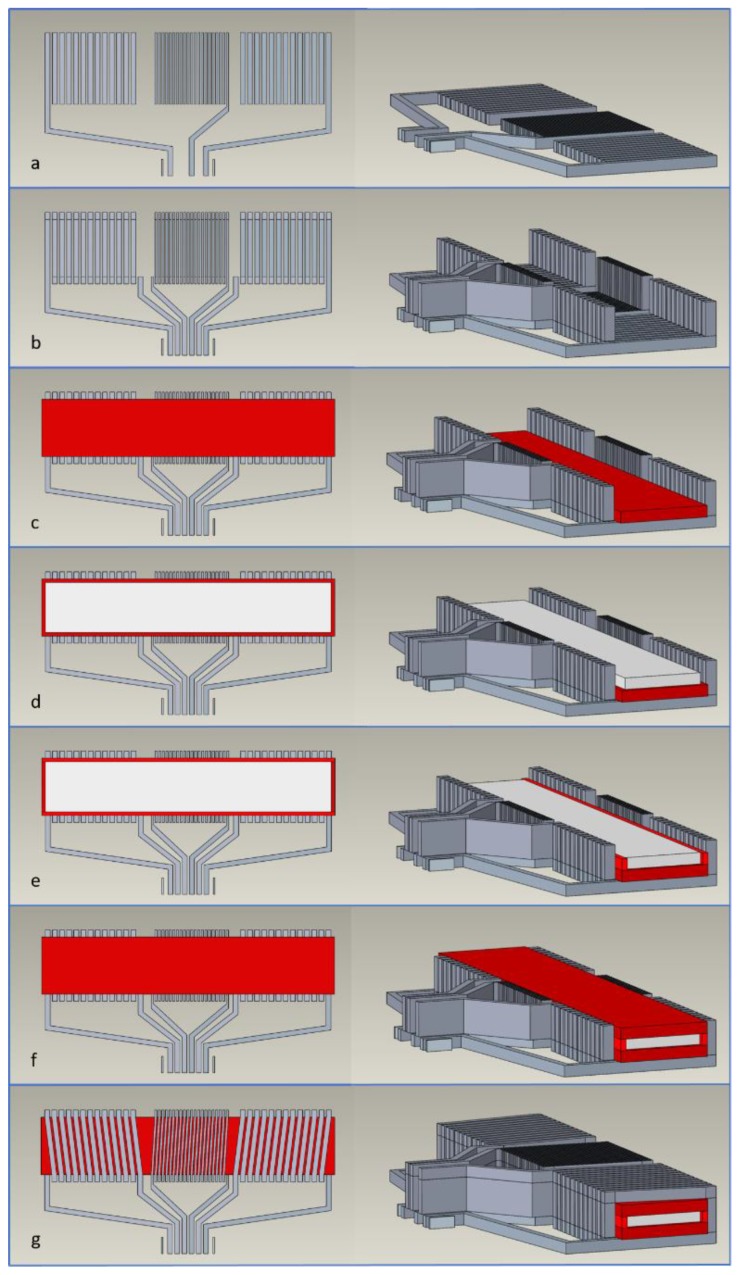
Schematic representation of the sensor after each fabrication step: (**a**) First step: printing the bottom conductive layer; (**b**) Second step: printing the conductive vias; (**c**) Third step: printing the bottom insulating layer; (**d**) Fourth step: the mounting and attachment of the magnetic core; (**e**) Fifth step: printing the insulating walls and (**f**) the top insulating layer; (**g**) Sixth step: printing the top conductive layer.

The sensor is structured with five main layers: two conductive printed layers, two insulating printed layers and the magnetic core in the form of a ribbon. The step-by-step fabrication process is illustrated in Figure 2. The first layer on the substrate is composed of the bottom conductive layer, which is formulated with silver-based ink (Figure 2a). The next step is the printing of the conductive vias (Figure 2b). This makes it possible to overcome the thickness of the next layers, comprising both the magnetic core and the insulation. In addition, this print will create the desired continuity for the final conductive layer. The print that follows, is the bottom insulating layer (Figure 2c). This print covers the tracks under the magnetic core in order to prevent any possible short-circuit. The magnetic core is accurately placed and glued on to the surface of the lowest insulating layer (Figure 2d). The next print forms the insulating walls lateral to the magnetic core (Figure 2e) and the top insulating layer (Figure 2f). With this print, the insulation that surrounds the magnetic core is completed. The last print consists of the top conductive layer, which forms the solenoid coils and completes the sensor (Figure 2g).

A thermal treatment following each print provides the polymerization of the inks. The sintering of the silver is not achieved with this curing procedure due to the relatively low applied temperature. However, the ink solvent evaporates during this process resulting in contact between the silver flakes and the ink. For each of the first two steps, thermal treatment at 200 °C for one hour is applied. For the following printing steps, thermal treatment at 100 °C for two hours is also applied. In particular, an intermediate curing process was employed for the printing of the conductive vias (Figure 2b) due to its great thickness. A thermal treatment every three prints with a handheld electric heat gun facilitated the procedure.

A KLA-Tencor Alpha-Step D-120 Stylus (KLA Corporation, Milpitas, CA, USA) was used for measuring the thickness of the fabricated sensor. The average thickness of a fabricated batch of sensors, including the substrate of 127 μm, was measured at 300 μm, while the average thickness of 34 consecutive printed layers of conductive ink was measured to be 100 μm. This height difference can be explained by the fact that the printing of additional layers forces the lower layers to lose their initial limits. Occasionally, this may cause short-circuits between the conductive tracks. The applied force of the pad compresses the bottom layer stack. Therefore, by growing in height (thickness), the printed layers obtain a trapezoidal shape. Thermal treatment between each layer can prevent this deformation.

Following the fabrication process, each sensor is checked for short-circuits between the solenoid coils and the magnetic core. In addition, the electrical characteristics of the sensor coils are measured at this stage. The average inductance value for the excitation coils is 0.75 ± 0.08 µH, while the average for the sensing coil is 1.25 ± 0.17 µH, at 100 kHz measuring frequency. The resistance of the coils is also measured. The average measured resistance value for the excitation coils is 21.7 ± 3.15 Ω, while the average for the sensing coil is about 65 ± 9.75 Ω. The high resistivity of the conductive inks is the reason high resistance values are obtained, especially in comparison with windings made from bulk copper or silver. The average resistivity of the “SpectraZ 14” conductive inks was found to be 40 times the resistivity of pure silver.

A measuring bench with a Helmholtz coil configuration was used for the characterization of the sensor. At an equal distance between the coils and the centre of the formed cylinder, the sensor was placed at a flat (not curved) position longitudinally to the magnetic field lines. No capacitors were employed for the excitation or the output tuning of the sensing coil. The sensor was connected with the PCB board via a zero-force insertion connector with 6-pin, 1 mm pitch and top-contact. This connector provides simplified connectivity between the sensor and the coil excitation and measurement devices.

In this paper, only the flat position of the fluxgate sensor is considered, as it has previously been successfully tested in a curved position. Initial tests using Helmholtz coils to produce a uniform linear magnetic field show that there is a deviation to the conformal flat sensor measurements when the sensing coil starts to bend. A full observation of the sensor in curved positions was not performed in this study. For this, a specific measuring setup design would be required, to allow optimal magnetic field shape control and the appropriate electrical connection of the sensor. The basic working principle of the sensor is expected to be the same, independent of its flat or curved position. Three measurement cycles of the sensor were considered for observation. The average value and the standard deviation of these measurements are expressed in the figures in the form of error bars. The sensitivity of the sensor is calculated as the field-to-voltage transfer coefficient (dV/dB) for the second harmonic of the output of the sensor [29,30].

## 3. Characterization

### 3.1. Working Principle of the Sensor

The periodic modulation of the permeability of the soft magnetic core is responsible for the fluxgate effect. Both excitation coils simultaneously generate an AC magnetic field that periodically drives the magnetic core to saturation. When no external magnetic field is present, the induced voltage that can be measured on the sensing coil is symmetrical and only odd harmonics can be detected in the waveform. If an external magnetic field is applied in the sensing direction, the symmetry of the induced voltage is disturbed and thus harmonics begin to appear in the waveform [1,2]. The applied magnetic field can be measured through its proportionality to the second harmonic or further higher harmonics of the induced voltage on the sensing coil.

### 3.2. Performance of the Sensor

**Figure 3:**
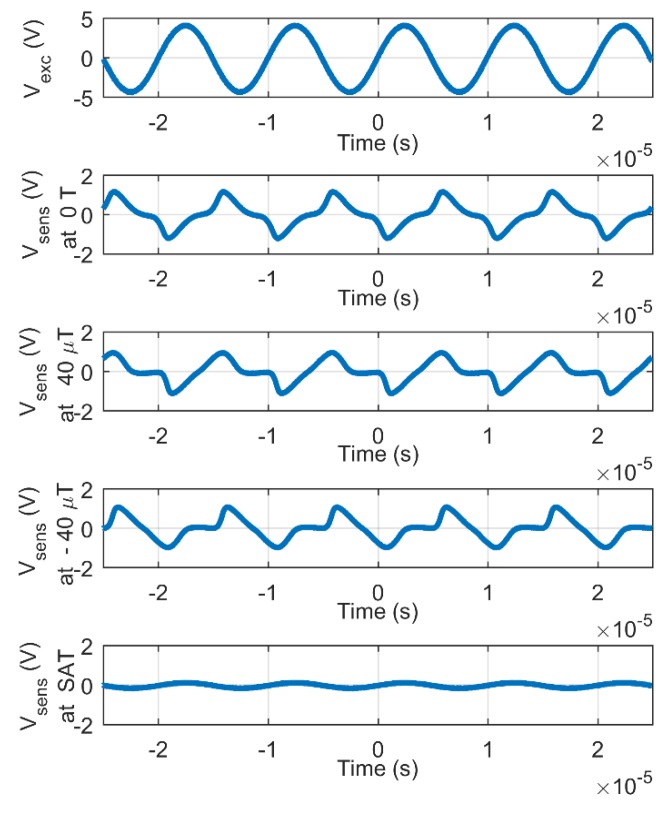
Waveforms of the fluxgate sensor with 700 mA p-p excitation current at 100 kHz: for the excitation coils (first signal) and for the sensing coil, respectively, at *B* = 0 T, *B* = +40 µT, *B* = −40 µT and at saturation.

Figure 3 presents typical sensor waveforms with 700 mA p-p excitation current at 100 kHz.

The output of the sensor comprises positive and negative peaks that are superimposed to a sinewave. Faraday’s law provides the expression for the induced voltage:

(1) $$V_{ind}=-\frac{d\Phi }{dt}=\frac{d\left(NA\mu_{0}\mu_{r}H\right)}{dt}=\mu_{0}N\left(HA\frac{d\left(\mu_{r}\right)}{dt}+A\mu_{r}\frac{d\left(H\right)}{dt}+H\mu_{r}\frac{d\left(A\right)}{dt}\right)$$

where Φ is the magnetic flux that depends on the number of turns of the sensing coil *N, H* is the magnetic field, $\mu_{r}$ is the permeability of the magnetic core, and *A* is the cross-section area of the magnetic core. The constant $\mu_{0}$ corresponds to the permeability of vacuum. In this formula, the demagnetization effect is neglected. The first term of equation (1) is responsible for the fluxgate effect and is generally exploited by fluxgate sensors. The second term corresponds to the induction effect and is principally exploited by search coil sensors. The third term is mainly exploited by sensors where the cross-section area *A* is time-dependent, such as rotary coil sensors. However, the magnetostriction of the magnetic core is generally negligible in fluxgate sensors and the last term of equation (1) can be neglected.

**Figure 4:**
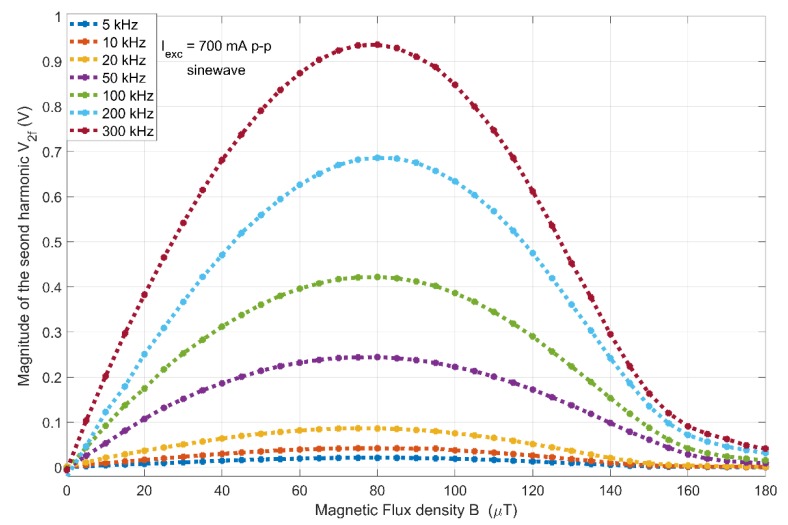
Magnitude of the sensor’s second harmonic response to the applied magnetic field at 700 mA p-p for 5–300 kHz excitation frequency range.

In this study, the second harmonic principle has been used to examine the main characteristics of the sensor. The magnitude of the sensor’s second harmonic response (V_2f_) to a varying magnetic field (B) from 0 to 200 µT is shown in Figure 4. The applied excitation current is of 700 mA p-p with frequencies ranging from 5 to 300 kHz. The resulting linear range of the sensor is found to be approximately ±40 μT, with a linearity error <2% FS at 100 kHz.

**Figure 5:**
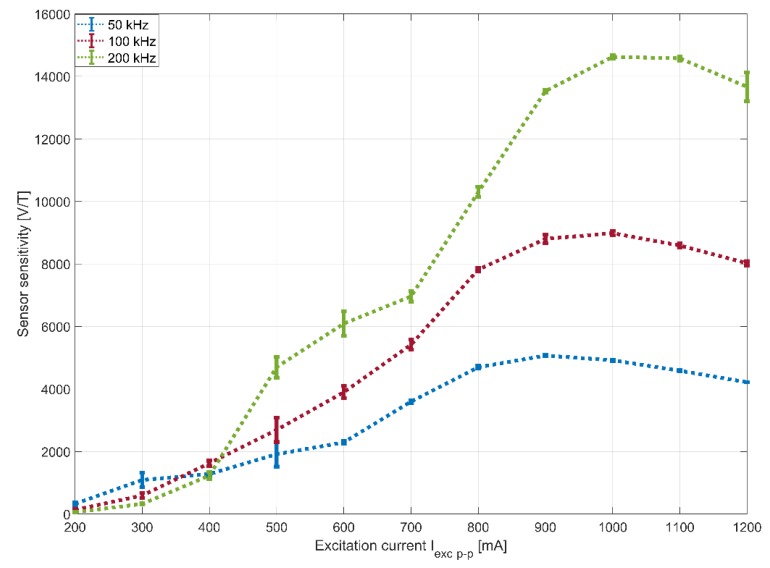
Sensor sensitivity as a result of excitation current.

The resulting sensitivity curves are presented as a function of the excitation current in Figure 5. The maximum sensitivity is measured at 14,620 V/T at 200 kHz for 1000 mA p-p excitation current, which is among the highest reported for micro-fabricated sensors in the literature [3,7,29,30]. Here, it can be observed that the sensitivity of the sensor increases at higher frequencies. Furthermore, the sensitivity depends on the excitation current magnitude, climbing to a certain limit with increasing current. According to [3], these maximum values are shifted at higher frequencies due to eddy current losses in the magnetic core. When applying excitation currents of a lower magnitude, the sensitivity is measured to be slightly higher at lower frequencies. This may result from the temperature dependence of the static or dynamic properties of the magnetic core such as permeability, partial saturation and skin effect. The inhomogeneous increase of temperature along the core may result in inhomogeneous magnetic properties and a slight change of behavior. In addition, parasitic coupling effects can affect the behavior of the sensor. A more detailed explanation of this phenomenon requires further investigation.

From the error bars, information indicating the stable operating points of the sensor can be extracted. The sensor becomes more stable the closer it reaches the maximum values of each curve. This happens at a relatively high current, which is required to saturate the thin magnetic core with the designed 13-turn excitation solenoid coils. Compared with other micro-fabricated fluxgate sensors [3,4,6,7,11,12], the applied excitation current is relatively high. Consequently, the consumption of such sensors, if operated permanently with sinusoidal excitation, is high. Interestingly, this shows the capability of the ink-based printed solenoid coils to drive high currents. The considerably high resistivity of the conductive inks (40 times higher than that of pure silver) causes a non-negligible temperature increase in the sensor due to the Joule effect.

Moreover, the noise of the sensor was measured with an HF2LI 50 MHz Lock-in Amplifier (Zurich Instruments Ltd., Zurich, Switzerland). The noise was found to be 10 nT/$\sqrt{\mathrm{Hz}}$ at 1 Hz, using the Lock-in Amplifier as a spectrum analyser.

### 3.3. Temperature of the Sensor

**Figure 6:**
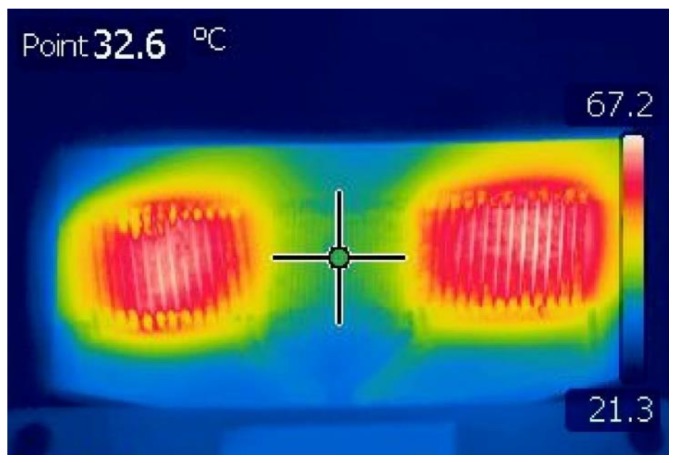
Temperature distribution of the sensor with 700 mA p-p excitation current at 100 kHz.

This section examines how the temperature of the sensor varies as a result of the excitation current. The temperature distribution of the sensor was measured using a FLIR T365 (FLIR Systems, Inc., Wilsonville, OR, USA) infrared camera, with ambient temperature at 20 °C. The sensor was placed on a 3D printed flat substrate with a thickness of 5 mm, composed of acrylonitrile butadiene styrene (ABS) filament. No passive or active cooling mechanisms were employed. Infrared measurements are based on the emissivity of the materials. In Figure 6. an infrared photograph with the temperature distribution on the sensor is presented. The temperature was measured with 700 mA p-p excitation current at 100 kHz. According to this figure, the temperature in the middle of the sensor, at the centre of the magnetic core is measured to be 32.6 °C, and the highest temperature that is dissipated from the excitation coils is 67.2 °C. The measured temperature from the excitation coils is dominant in this figure and corresponds to the surface temperature of the coils. On the other hand, the measured temperature in the middle of the sensor corresponds to that of the magnetic core, and thus can be considered homogenous for the entire thickness of the sensor at this particular point. Both measurements help to understand the way that the excitation current and frequency influence the sensor. In addition, the imperfections of the printing technique can be observed, since the excitation coils are not symmetrically heated by applying the same excitation current.

**Figure 7:**
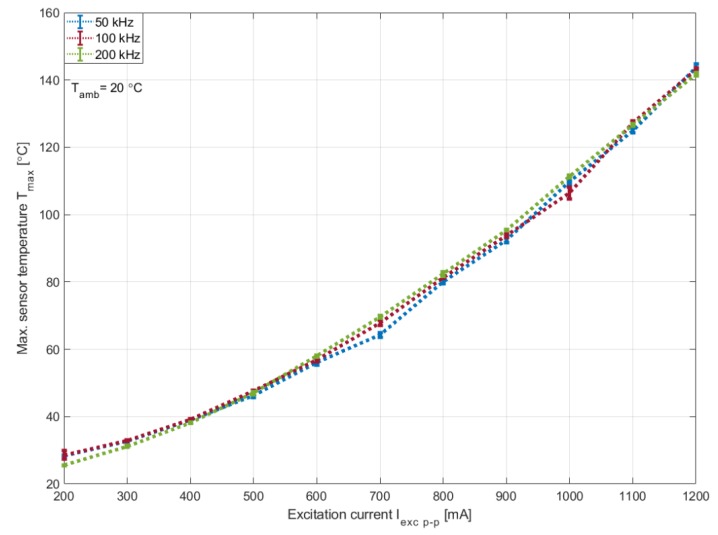
Maximum measured temperature of the sensor as a function of the excitation current.

**Figure 8:**
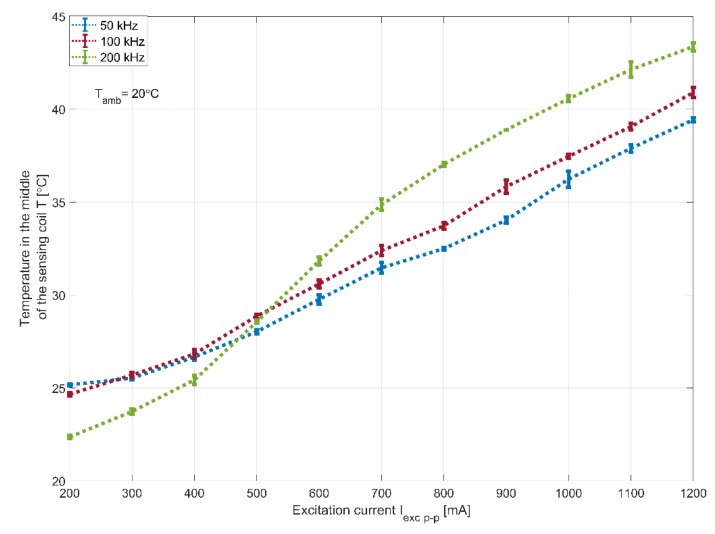
Measured temperature at the centre of the magnetic core as a function of the excitation current.

Furthermore, temperature measurements were taken using the infrared camera as a function of the excitation current for 50, 100 and 200 kHz. Figure 7 presents the maximum temperature that corresponds to the excitation coils and demonstrates where the curves for the different excitation frequencies are almost identical and their independence to the excitation frequency. Figure 8, however, presents the temperature measured in the middle of the sensing coil. These curves have a similar form to the sensitivity curves of Figure 5. At low currents, the measured temperature is higher for lower frequencies. However, as the excitation current increases, higher temperatures are recorded for higher frequencies. This reinforces previous assumptions regarding the increase in losses in the core under such conditions.

## 4. Conclusions and Discussion

In this paper, a flexible fluxgate sensor with solenoid coils fabricated with the pad-printing technique is presented. The sensor’s linear measuring range is ±40 µT with 2% full-scale linearity error, at 100 kHz excitation frequency. The highest measured sensitivity of the fabricated sensor was found to be 14,620 V/T at 200 kHz for 1000 mA p-p excitation current, which is among the highest reported, when compared with other micro-fabricated fluxgate sensors. The magnetic noise is 10 nT/$\sqrt{\mathrm{Hz}}$ at 1 Hz. This rather high noise level was expected, since this is a known characteristic of sensors with a bar design, which acts as a linear transformer. Lower noise levels could be obtained by employing a closed magnetic loop configuration such as ring-core or racetrack sensor design.

The flexibility of the sensor provides new possibilities in sensing curved magnetic fields. In addition to flexibility, the thinness and compact size of the sensor permits ease of integration in confined spaces. A possible use of the sensor would be to evaluate curved magnetic fields similar to those generated from a linear current-carrying cable. The evaluation of curved magnetic fields using flexible sensors could be a promising subject of a future study.

Printed solenoid coils with 250-µm-wide and 100-µm-high conductive tracks have been obtained. The sufficient current could be fed into these tracks to saturate the magnetic core. Despite the high resistivity of the “SpectraZ 14” conductive silver-based inks, which is 40 times higher than that of pure silver, the resulting thermal increase has been carefully managed. Further development of the inks would improve their conductivity.

The pad-printing technique is a promising complementary microfabrication method. The realization of prints on different kinds of material substrates is now possible. The substrates are able to have non-flat forms, and prints on convex and concave surfaces have already been realized. Consequently, this printing technique could be used as a post-process fabrication method. In addition, this technique allows the combination of printed components with a thin soft magnetic core, an otherwise non-printed component. This study also presents the possibility of the creation of low-cost transformers and actuators with the use of this micro-printing technique.
